# Delayed Diagnosis of Unilateral Mandibular Condylar Fracture in a Posterior Edentulous Patient

**DOI:** 10.1155/2021/5579236

**Published:** 2021-05-16

**Authors:** Sahani Anupama, Pilana Vithanage Kalani Shihanika Hettiarachchi

**Affiliations:** Department of Oral Medicine and Periodontology, Faculty of Dental Sciences, University of Peradeniya, Sri Lanka

## Abstract

**Background:**

Fractures of the mandible are common in elderly patients, and among them, condylar fractures are the most frequent type. A change in occlusion is the most common physical finding in patients with fractures of the mandible. Therefore, it is challenging to identify mandibular fractures in posterior edentulous patients due to the lack of posterior occlusal contacts. It is crucial to do radiological investigations in such patients to exclude fractures. *Case Presentation*. This article describes a case of delayed diagnosis of a unilateral mandibular condylar fracture for a week's duration and treating the condition as temporomandibular pathology in a posterior edentulous, 52-year-old patient.

**Conclusion:**

This clinical case highlights the importance of radiological investigations and occlusal analysis for early diagnosis of condylar fractures, particularly in posterior edentulous patients, lacking posterior occlusal contacts.

## 1. Introduction

Among facial bone fractures, mandible fracture has the highest incidence next to nasal bone fractures [1]. Out of mandibular fractures, condylar fractures are the most frequent accounting for between 29% and 52% of all mandibular fractures [2]. Binding of the mandibular ramus with high stiffness and mandibular condyle head with low stiffness can be considered the reason for the high incidence of mandibular condylar fractures [1].

The main causative factor for maxillofacial fractures including condylar fractures is road traffic accidents, violence, industrial hazards, falls, and sports accidents. Falls are the main etiology for maxillofacial trauma in children and geriatric patients, and falls are mostly responsible for mandible fractures in the latter group [3, 4].

Condylar fractures are most commonly missed on clinical examination. Therefore, condyle fractures are diagnosed with the help of both clinical and radiological assessment [5]. Clinical signs such as jaw deviation, mouth opening limitation, malocclusion, and edema of the preauricular region can be indicative of fractures of the condylar process [6]. A change in occlusion is the most common physical finding in patients with fractures of the mandible including condylar fractures [5]. But it is difficult to rely on this feature in patients who lack posterior occlusal contacts due to posterior edentulousness. Therefore, in these kinds of patients, we need to rely on radiographic evaluation to exclude condylar fractures.

Nogami et al. found that the incidence of fracture in the condylar region was significantly lower in patients with contacted molars in the maxilla and mandible as compared to affected patients with noncontact molars and suggested that contacted molars in the maxilla and mandible have an influence on condyle fractures in elderly individuals [4].

A mandibular condyle fracture may cause long-term complications such as mandibular growth and functional disorders and chronic temporomandibular joint (TMJ) complications such as TMJ ankylosis especially in children [1]. Therefore, to prevent these complications, early recognition and management of condylar fracture are paramount. This report describes the case of delayed diagnosis of a unilateral condylar fracture in a posterior edentulous 52-year-old patient, highlighting the importance of initial radiological examination in such cases.

## 2. Case History

A 52-year-old female patient was presented to the Oral Medicine Clinic of Dental Teaching Hospital, Peradeniya, with chief complaint of pain on the right-side TMJ region for 1-week duration. Patient had a history of fall a month ago due to an epileptic attack and hitting an object in the right-side TMJ region during the fall. The patient had presented to a local hospital initially and was treated with a course of analgesics which had been ineffectual; therefore, the patient sought medical advice from a specialist centre. Past medical history revealed that the patient was under antiepileptic medication for epilepsy for 30 years. She had no known allergies.

Examination revealed tenderness of the right-side TMJ region and slight restriction of mouth opening. No deviation of the mandible was observed. Intraoral examination showed that the patient was partially edentulous and had no posterior occlusal contacts. Out of the molar teeth, only 37 and 47 were present. Laceration or hematoma was not observed on the mucosa. No other significant findings were observed in the oral cavity.

**Figure 1:**
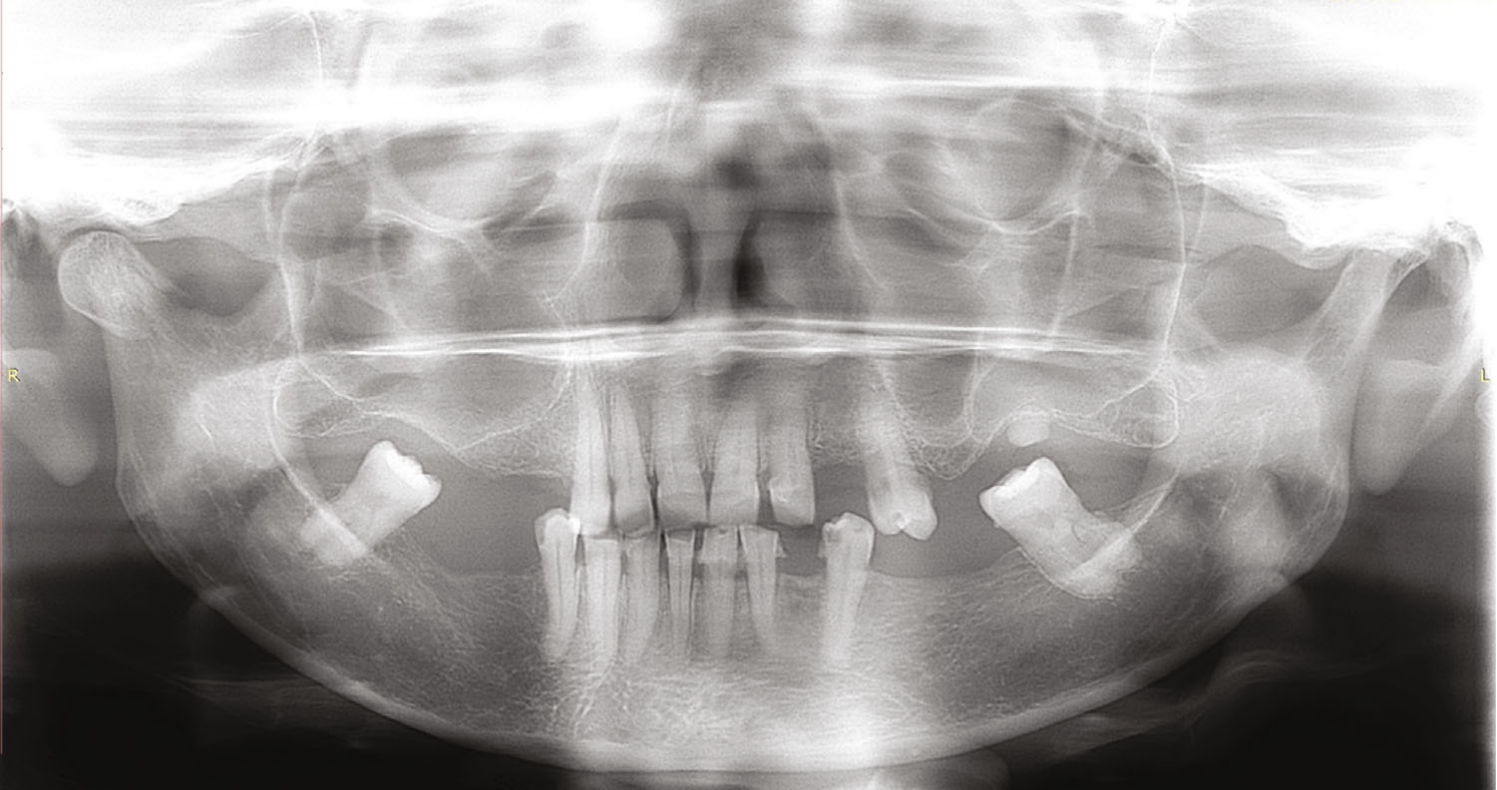
Panoramic radiograph demonstrating the condylar fracture on the right side.

Based on the clinical picture, TMJ disorder was suspected at the outpatient department. Therefore, the patient was initially treated with analgesics, muscle relaxants, and heat fermentation of the said region. Also, advice was given on self-care and to take a soft diet, and a review appointment was scheduled after a week. However, as the patient was symptomatic and there was no improvement of the condition, she has sought treatment from our clinic, where she was investigated with a panoramic radiographic examination. This revealed a fracture of the right-side condylar neck (Figure 1). The patient was referred immediately to the oral and maxillofacial unit for further management.

## 3. Discussion

Fractures of the mandible occur with a greater frequency in the elderly, and fractures of the condylar process of mandible are common injuries that account for 20-62% of all mandibular fractures [6]. The incidence of mandibular condylar fractures is higher in males compared to females [2, 4]. On the contrary, in the present case, the patient was a female.

The etiology for the condylar fracture documented in this case mirrors the existing literature in which accidental falls were the commonest cause [3, 4]. Also, the etiology of mandibular fractures is different between males and females; females had a higher incidence of mandibular fractures due to falls while males had a higher incidence due to road traffic accidents [7]. This fact was evident in the present case where the patient was female and had an accidental fall due to an epileptic attack.

Studies suggest that patients with epilepsy treated with antiepileptic drugs may be at an increased risk for bone disease including changes in bone turnover, osteoporosis, alterations in bone quality, and most importantly fractures [8]. However, mandible fracture cases have rarely been reported [9]. This case report belongs to that category. It is a widely known fact that epileptic seizures may be associated with fractures and dislocations either owing to the falls associated with the seizure or, indeed, owing to the mechanism of the seizure itself. In this case, it was due to the fall associated with the seizure. In addition, it is well established that antiepileptic drugs increase fracture risk by an average of twofold to sixfold [9]. In the present case, as the patient was on antiepileptic drugs for a long period of time, it can be speculated that this may have been one of the reasons for the fracture to have occurred.

There are many reasons which can contribute to condylar fractures in the elderly. Werning et al. showed that osteoporosis is an independent risk factor for the development of maxillofacial fractures in the elderly [10]. Osteoporosis can be defined as a common metabolic bone disease characterized by low bone mass, microarchitectural weakening leading to bone fragility, and increased fracture risk. Osteoporosis predominantly affects elderly women as the rate of bone loss increases after menopause in women. Women show an estrogen-related bone loss commencing at menopause, mainly in trabecular bone, followed by a slower loss of both trabecular and cortical bones 4–8 years later [11]. It is a known fact that osteoporosis results in reduced jaw bone mass as well as alterations of the mandibular structure, especially of the inferior border [12]. Also, antiepileptic drugs have also been shown to cause osteoporosis and bone fractures in the absence of vitamin D deficiency [9]. Hence, in the present case, the patient may have had osteoporosis as the patient was a postmenopausal women and was using antiepileptic drugs. This may have contributed to the condylar fracture.

Bisphosphonates are the most widely used class of antiresorptive drugs. Bisphosphonate use plays a primary role in the management of metastatic bone diseases as well as osteoporosis. In patients suffering from metastatic bone diseases, bisphosphonates provide significant protection against skeletal complications, in particular against fractures. But one of the serious side effects of bisphosphonates can be considered the bisphosphonate-related osteonecrosis of the jaws (BRONJ). This condition can even lead to pathological fractures of the mandible [13]. But the above factors may not have contributed to the condylar fracture in the current case as the patient was not on bisphosphonates.

Nogami et al. found that incidence of fracture in the condylar region was significantly lower in patients with contacted molars in the maxilla and mandible as compared to affected patients with noncontacted molars and advanced the hypothesis that the presence of contacted molars may work as “a vertical stop” and be a protective element for the condyle [4]. Hasegawa et al. showed that the risk of condylar fractures was significantly higher in patients without occlusal support or mandibular third molars [14]. This was highlighted in this case where the patient had no posterior occlusal contacts as out of all the molars only 37 and 47 were present.

Zhou et al. examined the influence of unerupted mandibular third molars on the incidence of condyle fractures and found a significantly higher incidence of fractures in the condylar region in patients without than in those with unerupted third molars [2]. This case agrees with this finding as the mandibular third molars were absent in this patient.

The patient's history and the clinical examination are helpful in the diagnosis of condylar fractures but radiographs are probably the most important aid as condylar fractures are most commonly missed on clinical examination [5]. This was evident in this case as the diagnosis of condylar fracture was missed at the initial presentation due to the lack of clinical signs, and also, initially, a TMJ disorder was suspected. This was mainly due to the posterior edentulousness of the patient, and as a result, alterations in the occlusion were not evident. Therefore, the authors would like to emphasize the importance of radiological evaluation in such patients at the initial presentation to exclude condylar and/or mandibular fractures.

Routine plain radiological investigations that aid in the diagnosis of condylar fracture include oblique views, posteroanterior view, and Towne's view [5]. Computed tomography (CT) is considered a gold standard for the radiographic evaluation of fractures of the mandibular condyle process. It can be used to evaluate both bony and soft tissue changes, and multiplanar evaluation is an added advantage. However, the routine use of CT for mandibular fractures is not justified due to the high cost and increased radiation exposure [6]. Panoramic radiograph is the most comprehensive view possible with a single film and provides good visualization of the entire mandible including the condylar region; therefore, it is commonly used by many clinicians as an ideal screening view for mandibular fractures [5, 6]. In the present case as well, panoramic radiograph was used as the radiological diagnostic aid in which the condylar fracture was identified.

Complications of mandibular condylar fractures includes nonunion, malunion, malocclusion, TMJ derangement, traumatic arteritis, ankylosis of TMJ, condyle resorption, growth disorders mainly in young patients, and facial asymmetry [1]. To prevent these, it is important that the condylar fractures are diagnosed and managed early.

## 4. Conclusion

Although condylar fractures are common in elderly patients, they are frequently overlooked by the clinicians. In the geriatric population, the dental elements tend to decrease in number, and posterior edentulousness is a common presentation among them. In such patients presenting with pain in the TMJ region, it is eminent to not only rely on clinical features but also carry out radiological evaluation to exclude condylar fractures and/or mandibular fractures at the initial presentation itself. This will aid in early identification and management of condylar fractures, preventing the possible complications.
